# Prediction of Target-Drug Therapy by Identifying Gene Mutations in Lung Cancer With Histopathological Stained Image and Deep Learning Techniques

**DOI:** 10.3389/fonc.2021.642945

**Published:** 2021-04-13

**Authors:** Kaimei Huang, Zhiyi Mo, Wen Zhu, Bo Liao, Yachao Yang, Fang-Xiang Wu

**Affiliations:** ^1^ Key Laboratory of Computational Science and Application of Hainan Province, Haikou, China; ^2^ Key Laboratory of Data Science and Intelligence Education, Hainan Normal University, Ministry of Education, Haikou, China; ^3^ School of Mathematics and Statistics, Hainan Normal University, Haikou, China; ^4^ School of Data Science and Software Engineering, Wuzhou University, Wuzhou, China; ^5^ Division of Biomedical Engineering, Department of Mechanical Engineering, Department of Computer Science, University of Saskatchewan, Saskatoon, SK, Canada

**Keywords:** lung cancer, targeted therapy, pathological images, convolution neural network, residual network

## Abstract

Lung cancer is a kind of cancer with high morbidity and mortality which is associated with various gene mutations. Individualized targeted-drug therapy has become the optimized treatment of lung cancer, especially benefit for patients who are not qualified for lung lobectomy. It is crucial to accurately identify mutant genes within tumor region from stained pathological slice. Therefore, we mainly focus on identifying mutant gene of lung cancer by analyzing the pathological images. In this study, we have proposed a method by identifying gene mutations in lung cancer with histopathological stained image and deep learning to predict target-drug therapy, referred to as DeepIMLH. The DeepIMLH algorithm first downloaded 180 hematoxylin-eosin staining (H&E) images of lung cancer from the Cancer Gene Atlas (TCGA). Then deep convolution Gaussian mixture model (DCGMM) was used to perform color normalization. Convolutional neural network (CNN) and residual network (Res-Net) were used to identifying mutated gene from H&E stained imaging and achieved good accuracy. It demonstrated that our method can be used to choose targeted-drug therapy which might be applied to clinical practice. More studies should be conducted though.

## Introduction

Lung cancer has the highest morbidity and mortality worldwide, and it became the main cause of death in China, especially for males (1). According to different biological factors and clinical presentations, lung cancer could be further divided into two sub-types: small cell lung cancer and non-small cell lung cancer (2). Non-small cell lung cancer takes up nearly 80 to 85% of all lung cancers, and small cell lung cancer takes up only 15 to 20% (3). Small cell lung cancer is a kind of invasive and malignant neuroendocrine tumor however sensitive to chemotherapy and radiation therapy. Therefore, the therapy of small cell lung cancer is relatively effective and with good prognosis (4). However, most lung cancers are non-small cell cancers, which contains lung squamous cell carcinoma (LUSC), lung adenocarcinoma (LUAD), and large cell carcinoma, which are significantly less sensitive to chemotherapy and radiotherapy compared to small cell lung cancer (5, 6). Patients with squamous cell carcinoma do not well response to anti-tumor drugs therapy because of intolerable toxic complications (7, 8). However, there are genetic abnormalities carried by patients with adenocarcinoma who respond well to targeted-drug therapy (9, 10), such as those carry EGFR mutations or ALK rearrangements (11). Studies have shown that the prognosis of patients is strongly associated with certain histopathological features (12, 13). The efficacy of targeted-drug therapy depends on the stage of cancer by pathological diagnosis, therefore it might enhance the patients’ quality of life and prognosis by identifying gene mutations (14, 15).

Although many techniques are approved to have significant advantages on diagnostic imaging, visual inspection of histological stained slice is still considered as “gold method” for tumor diagnosis. But, the diagnostic accuracy and treatment plan generally on the basis of the results of biopsy study. It requires well-experienced pathologists who can confidently identify the changes of cell morphology and corresponding tumor stage of lung cancer by visual inspection of pathological images (16, 17). Currently, the pathologists can determine the histological stage of tumor by looking at stained slice under microscope. However, the pathological report might be subject to individual bias and staining technique (18).Therefore, cancer diagnosis usually requires several pathologists to evaluate the same stained slice in order to increase accuracy of diagnosis which is time consuming and costly. Furthermore, poorly differentiated tumors or those in advanced stage also brings challenges to make a reliable diagnosis, thereby, computer aided assessment is recommended for diagnosis and designing therapy plan.

Deep learning means have been used in the medical field for lots of years (19), which can save time and receive reliable diagnosis, especially in image assessment. In oncology field, it has already gained approval for better efficiency, accuracy, and consistency diagnosis (20). Compared to experienced pathologist, it has advantage of identifying tumor region by image segmentation, sub-type classification, as well as predicting the disease prognosis (21). Pegah Khosravi et al. established an independent frame according to Convolutional Neural Networks (CNN), to classify the histopathological slices from different types of cancer, and gain good results (22). Jakob Nikolas Kather et al. reported that deep residual learning can predict microsatellite instability directly from hematoxylin-eosin staining slice (23). Moreover, the study conducted by Zizhao Zhang et al. came up with a new artificial intelligence-driven of histological staining slice diagnostic approach which solved the problem of interpretable diagnosis (24). Nicolas Coudray et al. downloaded full-slice images from the cancer genome atlas, they annotate tumor region on one slice, and remaining slices were divided into training set or validation set. The data from training set was trained by deep convolutional neural network (inceptionv3) and finally automatically and accurately classified data into lung adenocarcinoma, lung squamous cell carcinoma, or normal lung tissue (3). Those studies demonstrated that deep learning methods can achieve relatively good results in analyzing the pathological images of patients.

Currently, there are different types of cancer therapy, which range from the traditional radio-logical and broad spectrum chemotherapy, to targeted-drug therapy. “Target therapy” is to apply advanced technology to accurately deliver drugs to the tumor region in order to specifically eliminate malignant cells without damaging normal tissue cells. The basis of “target therapy” is aiming to design an individualized treatment plan targeting to specific malignant cells by applying current knowledge of cancer biology and pathogenesis at the cellular and molecular levels. Therefore, it is necessary to develop novel machine learning methods for diagnosis and design treatment plan which might increase the efficiency, accuracy, and consistency of diagnosis.

This paper aimed to predict the mutated genes which are potential candidates for targeted-drug therapy by developing a novel algorithm according to convolutional neural network for lung cancer. In this study, we used hematoxylin-eosin (H&E) stained pathological slice of lung cancer which were downloaded from the TCGA, thereby, deep convolution Gaussian mixture model DCGMM was used to perform color normalization. Convolutional neural network (CNN) and residual network (Res-Net) were employed for training data. The average probability of the bio-markers of lung cancer was received through the model, with the highest accuracy rate of the MET which was reached 86.3%. It provided an approach to develop effective targeted therapy on basis of mutant genes of lung cancer, however, it need further studies to evaluate the effectiveness and reliability of designed model before applying to clinical practice.

## Materials and Study Framework

### Data Set Preparation

We downloaded H&E histopathology images of lung cancer from the Cancer Genome Atlas (TCGA) (https://portal.gdc.cancer.gov/repository/) as our data set. Then we used cBioportal (http://www.cbioportal.org/index.do) to download H&E image SNP data, Cancer Genomics Portal provides a visualization tool for analyzing of cancer gene data. cBioPort was used to perform molecular and cytological studies for genetic, epigenetic, gene expression, and proteomics research. The clinical data of H&E image lung cancer patients were downloaded from the International Cancer Genome Collaborative Group (ICGC) (https://dcc.icgc.org/).

We used the python package OpenSlide to analyze the histopathological images as SVS format. The experienced pathologists examined the H&E stained image and identified the abnormal or suspected area which may have diagnostic significance. They also discarded low-quality slices which have blurred background or contained other tissues around, such as inflammatory cells, micro-vessels, microfibers and lymphoid, etc. Then cBioPortal was performed to identified potential mutant genes associated with lung cancer as bio-markers, such as AKT1, ALK, BRAF, FGFR1, FGFR2, HRAS, KRAS, MET, etc. Moreover, we labeled the H&E stained slice with the potential lung bio-markers as 1, otherwise, labeled as 0.

### Study Framework

**Figure 1:**
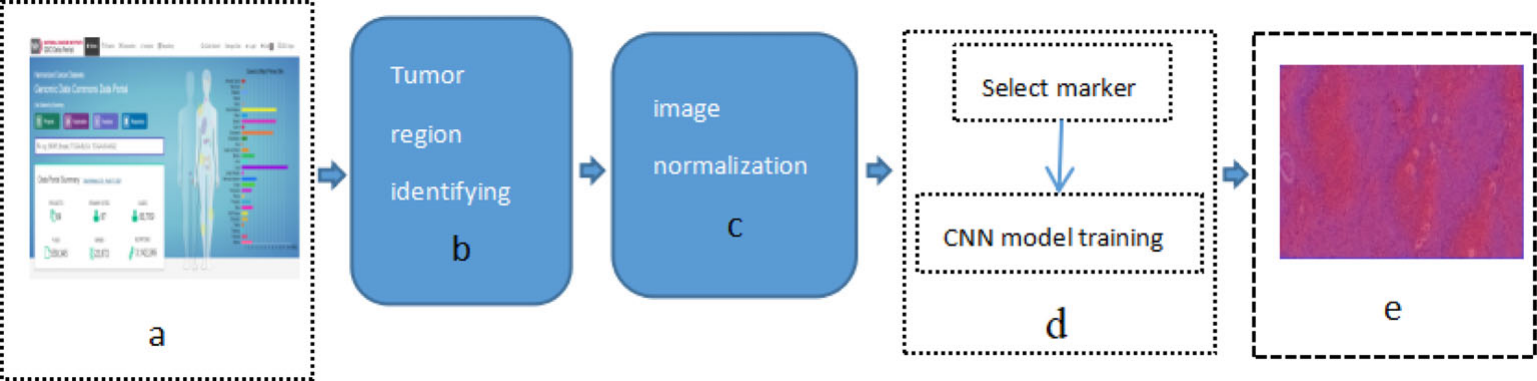
Study flow chart **(A)** Download images from The Cancer Genome Atlas database; **(B)** identify tumor areas; **(C)** color normalization; **(D)** biomarker recognition; **(E)** heat map.

This study applied previously designed framework, as show in **Figure 1** [Study flow chart (a) Download images from The Cancer Genome Atlas database; (b) identify tumor areas; (c) color normalization; (d) bio-marker recognition; (e) heat map], which is DeepIMLH to annotate the tumor region of lung cancer on H&E stained slices, thereby to identify mutated genes as the potential bio-markers for targeted-drug therapy of lung cancer. As shown below, this study included five steps. Firstly, 180 hematoxylin and eosin (H&E) stained whole slice images (WSI) of lung cancer were downloaded TCGA; secondly, the experienced pathologists annotated the tumor area on the H&E stained slices and WSI were further divided into tiles of 512*512 pixels window; thirdly, the performance analysis of image model is usually compromised to subjective bias due to many factors such staining technology and processing procedure, the quality of biopsy sample, etc. In order to prevent the potential bias, Gaussian mixture model was used for color normalization of H&E stained slices. Thereby, all the images were through subsequent model training. Fourthly, WSI contained mutant bio-markers of targeted therapy was annotated and further input for training by a new convolutional neural network (CNN) model combined with residual blocks. Finally, the trained tiles were classified and summarized to full slices for extraction of heat maps.

### Identify Tumor Areas

**Figure 2:**
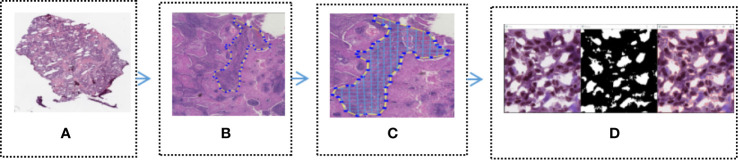
Tumor region recognition model **(A)** Full slice image downloaded from TCGA; **(B)** Professional pathologist annotated tumor region; **(C)** Segmented WSI with 512 * 512 sliding window; **(D)** Block noise reduction processing.

In order to identify the tumor area, experienced pathologists firstly annotated 180 H&E stained whole slide images (WSI) of lung cancers which were downloaded from the TCGA, as show in **Figure 2A** (Download images from TCGA). The experienced pathologist can annotate the boundary of tumor area, such as abnormal cell nuclei, cell shape under the microscope. The area surrounded by the blue-yellow circle in the **Figure 2B** (Professional pathologist annotated tumor region) was the boundary of tumor area. Before the CNN model training, the full slide image was divided into small pieces of the same size with a 512*512 pixel window, which was shown in **Figure 2C** (Segmented WSI with 512*512 sliding window). Downloaded WSI always have some background noise, since they came from different biopsy samples and have different background. In order to reduce significant interference in subsequent training, we remove the background noise, blank or large fuzzy areas. Python’s OpenCV was used to calculate the ratio of the area of blurred background of the tile over the total area of slice. The threshold was set in order to remove samples which is less than the threshold. It was shown as **Figure 2D** (Block noise reduction processing). Python’s OpenCV software package was also used to segment H&E stained slices. Finally, it was integrated into the new data-set and split into a training set and a verification set according to a 1:1 ratio.

### Color Normalization

H&E staining is a commonly used staining technique which are widely used in the tumor diagnosis and staging. However, this method might subject to inconsistent color fixing due to specimen preparation standard, staining technique, H&E reagents, the thickness of section, etc. The color difference of image is one of the most important factors in the training process of the CNN. Therefore, unsupervised deep convolution Gaussian mixture model (DCGMM) was applied to standardize the color of H&E stained slices.

**Figure 3:**
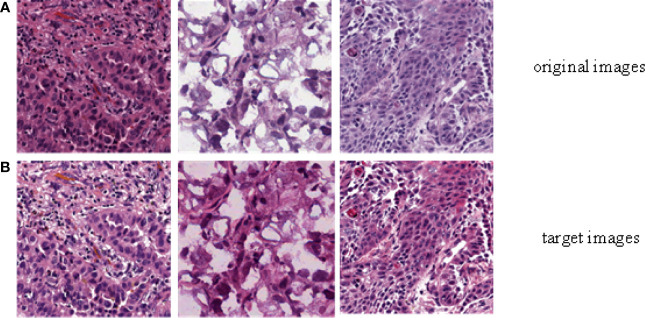
Color normalization model of H&E stained image. The image was normalized by the depth convolution Gaussian mixture model (DCGMM), **(A)** Original images, **(B)** Color normalized images.

In order to evaluate the efficiency of trained by DCGMM, these images should have consistent colors and unchanged features such as morphology, pixels, and structure after training, as show in **Figure 3** [Color normalization model of H&E stained image; the image was normalized by DCGMM, (a) Original images, (b) Color normalized images]. Color standardization is primarily based on the Gaussian distributed to average the original image, which can be converted to white and color. **Figure 3** was a frame diagram of color normalization of H&E stained slices. The upper three images in **Figure 3** were the original images without color normalization, the lower three pictures were those after color normalized. Comparing the original images with the target images, we can indicate the model only normalize the color of the image without changing in the size, pixels, and position of these images. Moreover, the model is unsupervised, it does not require any assumptions of the data or any label.

### Bio-Marker Recognition

In order to identify the bio-markers of lung cancer H&E stains, we used Tensorflow 2.0.0 software package to train the convolutional neural network model. The convolutional neural network can use back propagation to adjust the parameters of the convolutional neural network, extract the features of the image, and classify the images according to the extracted features. This is an effective gradient descent algorithm that can automatically update the weights during training.

We first input the processed lung cancer H&E stain slides and bio-markers to the input layer of the deep convolutional neural network, and then output them to the convolutional layer to extract image features. The convolutional layer is composed of 32 n*n convolution kernels. Secondly, enter the excitation layer containing the ReLu excitation function, and then enter the next hidden layers until all the features of the H&E stained slice are extracted. In order to improve the generalization ability of the network and accelerate the training of higher learning rates, we add a batch normalization layer to the convolutional neural network. However, this will increase the depth of the convolutional neural network, and cause the problem of gradient disappearance, which will slow down network training speed and classification accuracy. So we introduced a residual network with jump connections to solve the above problems. In addition, the optimization function and loss function used in the convolutional neural network are “adam” and “sparse categorical cross entropy” respectively. The output in here are slides with characteristics of different lung cancer bio-markers for targeted therapy.

### Heat Map

**Figure 4:**
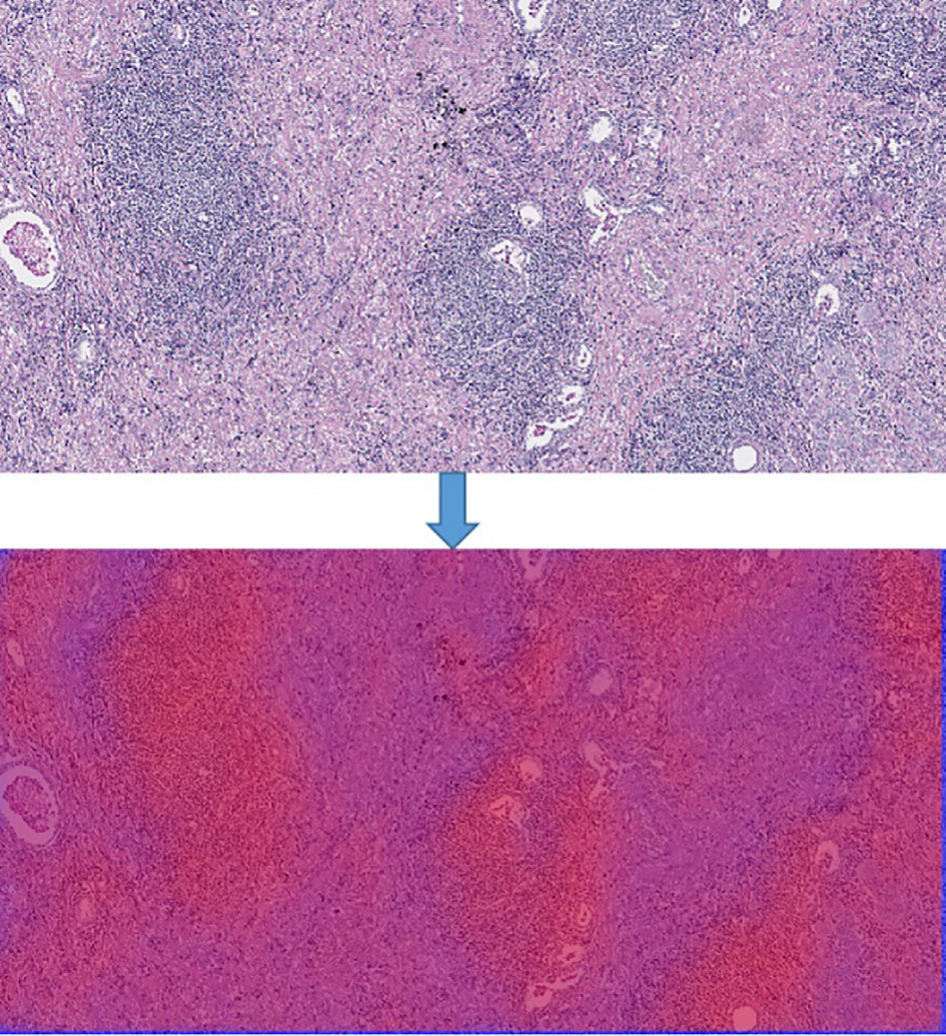
Heatmap of the tumor region applied in the CNN model by using TCGA dataset. The above image is the original image, the under image is the heat map.

In order to generate the heat map, we firstly scanned the whole slide images with 512*512 slicing windows of lung cancer bio-markers. Then the results of each slides were obtained through the CNN + Res-Net model by applying pixels of sliding windows. Moreover all the values of passed pixels were summed and calculate their average as bio-marker probability of targeted therapy for lung cancer. We used probability visualization to convert the probability of targeted therapeutic markers of pixels in to color values. The probability value was mapped in the range of (0, 1) to RGB color from pure blue color (0, 0, 255) to pure red color (255, 0, 0) linearly. Therefore the red color in the heat map indicated the higher the probability of bio-marker appearance, the blue color indicated lower the probability of bio-markers appearance. A WSI has multiple tiles, and each tile can predict corresponding result probability by the model. We integrate the results of all tiles through the fusion algorithm to obtain the final probability result of the corresponding WSI. Define the average probability of the first n windows as the recognition score. Predict the occurrence probability of lung cancer bio-markers by setting critical thresholds. Among them, those higher than the threshold are considered positive, otherwise they are considered negative. We use the highest value as a hyper-parameter and determined by cross-validation. The hyper-parameters of model were determined by providing hyper-parameter dictionary with using the Grid-Search-CV class in scikit-learn. The resulting heat map is shown in **Figure 4** (Heatmap of the tumor region applied in the CNN model by using TCGA dataset. The above image is the original image, the under image is the heat map). The up picture of **Figure 4** was the original H&E staining slice, and the under picture was the corresponding heat map.

## Results

In order to calculate the average probability of different bio-markers from lung cancer H&E full slices, we firstly downloaded 180 lung cancer H&E stained whole slide images from TCGA. The clinical characteristics of the patients were shown in **Table 1**. The experienced pathologists annotated the tumor area of H&E stained slices, segmented and noise reduction. Then we selected 1800 512*512 small blocks with good quality. Thereby 1,800 small pieces were randomly divided into two sets which were training set and verification set. Finally, we obtained the heat-maps of different bio-markers of lung cancer H&E full slices and the AUC (area under ROC curve) values by 2-fold cross-validation were by DeepLRHE model. The process was shown in **Figure 1**.

**Table 1:** Clinical Characteristics of the Study Patients.

Characteristics	Distribution of clinical information	number of patients
Gender	Male	99
	Female	81
Age at diagnosis	30–40	2
	41–50	13
	51–60	25
	61–70	63
	>70	77
Survival time (years)	0–1	25
	1–2	14
	2–3	16
	3–4	5
	>4	6
	Unknown	114
Cancer sub-type	LUAD	105
	LUSC	75

**Table 1** showed clinical information of patients whose H&E stained slices we downloaded from TCGA. The total patients number is 180, with 55% are female and 45% are men. The mean age was 68-year-old, and 42% of the patients were diagnosed as lung cancer at year over 70-year-old. Moreover, 13% of patient survived less 1 year with known cases. Among the 180 patients, 8% were diagnosed as lung adenocarcinoma, and 42% were lung squamous cell carcinoma.

### Performance Evaluation

By using the DeepIMLH model, we can get the average probability of different bio-markers from 180 lung cancer H&E stained slice. **Table 2** showed the accuracy of five frequently mutated genes which were predictable by the DeepIMLH model. The ALK, BRAF, and KRAS mutations might not been conceived due to imbalance of negative and positive samples. As shown in **Table 2**, the highest accuracy rate was MET with reached 86.3%, followed by FGFR1 83.2%, FGFR2 82.1%, HRAS 78.7%, the lowest was AKT1 of 72.3%.

**Table 2:** Accuracy of lung cancer biomarkers.

Cancer type	Bio-marker	Accuracy
Lung cancer	AKT1	72%
Lung cancer	FGFR1	83%
Lung cancer	FGFR2	82%
Lung cancer	HRAS	79%
Lung cancer	MET	86%

### ROC Curve of Bio-Markers

**Figure 5:**
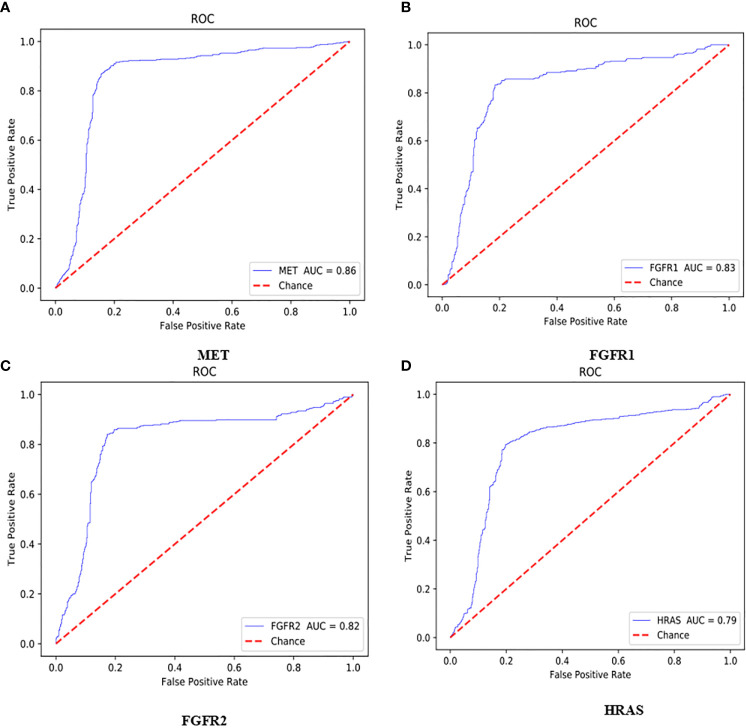
ROC curve with 512*512 image blocks by 2 times cross-validation, sub figures of **(A–D)** was the ROC curve of mutated gene MET, FGFR1, FGFR2, HRAS respectively.

The AUC value of the lung cancer bio-markers in this study was the average probability of each bio-marker on the lung cancer H&E stained full slice image. The ROC curve of each bio-marker was shown in **Figure 5** (ROC curve with 512*512 image blocks by two times cross-validation, sub figures of A–D was the ROC curve of mutated gene MET, FGFR1, FGFR2, HRAS respectively). The ROC chart was drawn with the false positive rate (FPR) as the X-axis and the true positive rate (TPR) as Y-axis. The area under the ROC curve was the AUC value. Only the ROC curves of MET, FGFR1, FGFR2, and HRAS bio-marker were shown here because of higher accuracy. Those markers may have effects on predicting the sensitivity to targeted therapy and disease prognosis. Targeted therapy has been rationally designed to suppress specific mutations and gain more effective clinical treatment.

As shown in **Figure 5A**, the AUC value of MET was reaching to 86%. The MET is proto-oncogene, by binding with its ligand hepatocyte growth factor (HGF) signaling pathway, it mediates wound healing and hepatic cell regeneration, and plays a critical role in the process of embryonic development. However, the non-regulated MET signaling pathway can cause abnormal cell proliferation, apoptosis, migration, even have potential for oncogenesis, malignancies. In non-small cell lung cancer, some of patients present MET mutation, including MET protein over expression, MET mutation or rearrangement, which lead to non-regulated downstream signaling pathway (25). Patients with MET mutation might not respond to therapy combining various targets or standard therapy with MET inhibitor. MET inhibitors have been went through clinical trials, the clinical data was promising now, which showed that MET mutation is a potential bio-marker to predict the response to target-drug therapy, as well as prediction of disease prognosis (26). Moreover, large clinical trial still ongoing to evaluate the predictive role in lung cancer therapy. In recent years, there have been endless researches on MET inhibitors. Among them, Volitinib, Tepotinib, and Capmatinib (also known as INC280) are the three drugs with relatively large research data (27).

As shown in **Figures 5B, C**, the AUC values of FGFR1 and FGFR2 were 83 and 82%, respectively. The fibroblast growth factor receptors (FGFRs) play a critical role in tumor genesis, cell proliferation, angiogenesis, cell migration, apoptosis, and survival. Early studies showed that inhibition of FGFRs can decrease cell proliferation and induce cell apoptosis in both *in vitro* and *in vivo* models with FGFR mutations, moreover, other studies also chose FGFRs as target for anticancer medical therapy. For an example, multiple kinase FGFR/vascular endothelial growth factor receptor (VEGR) inhibitor gained the promising results in breast cancer with FGFR/FGFR3 amplification (28). Moreover, early clinical trials also demonstrated that choice FGFR inhibitors may conquer the drug toxicity. To be specific, FGFR1, an oncogenic receptor tyrosine kinase (RTK), plays fundamental roles in the process of cancer prognosis. Under normal physiological condition, FGFR1 signaling pathway is triggered by many growth factors, leading to receptor dimerization and transphosphorylation, thereby, activated FGFR1 pathway leads to the down-streaming pathway including RAS/MAPK which is critical pathway in almost cancer development. FGFR1 is frequently amplified in lung cancer and is a latent curative target in many solid tumor as well. Clinical application of FGFR combined with FGF target remains unclear. FGFR inhibitors primarily target the cytoplasmic kinase domain, they also might target the extracellular ligand binding domain (29). Patients with FGFR mutation is potential a candidate for clinical trial for FGFR inhibitors. Clinical studies have shown the primary reason for the resistance of FGFR inhibitors may cause by bypass signal activation. The pharmacological or genetic mutation of FGFR induced autophagy; the mechanism remains unknown, which may involve both inhibition of ERK/MAPK pathway and decline.

As shown in **Figure 5D**, the AUC value of HRAS is 79%. The HRAS gene is an oncogene and a member of the RAS oncogene family, which also includes two other genes: KRAS and NRAS. The RAS gene codes for small membrane bound proteins and hydrolyze GTP and participates in the cascade of protein kinase, transmits signal to neclei (30). Activation of RAS gene family might convert those protogenesis to drive cancer development. These genes play important role in cell proliferation, differentiation, and apoptosis. Mutated RAS coded proteins are the key drivers in many cancers and the distribution of RAS mutation varies between the difference of somatic tumors. Studies have shown that if the RAS gene is mutated, Atradigen is one of the proven effective RAS inhibitor drugs in the world.

As shown in **Table 2**, the accuracy of AKT1 was 72%. The AKT1 gene encodes a serine/threonine protein kinase, which can be activated by extra cellular signals through a phosphatidylinositol 3-kinase (PI3K) (31). Currently, three members of the AKT family are found, naming AKT1/PKB α and AKT2/PKB β and AKT3/PKB γ respectively. AKT1 is a core factor in PI3K/AKT signaling pathway, and PI3Ks can specifically cause the three hydroxyl groups of phosphatidylinositol (PI). The production of second messenger inositol such as PIP3, PIP3 can promote AKT transferring to the cell membrane and can be activated by PDK1/PDK2. The activated AKT relocates in the cytoplasm, nucleus, or other organelles of the cell, large number of substrate proteins would be phosphorylated. Thereby, AKT signaling pathway can regulate multiple cell functions, however, the abnormally activated AKT signaling can cause tumorigenesis. For AKT1 gene mutations, everolimus and other mTOR inhibitors that have been on the market have good efficacy (32).

## Discussion

With the in-depth understanding of tumor molecular biology, targeted therapy has become one of the most popular treatment options for lung cancer. Some studies have showed the impact of targeted therapy in small cell lung cancer, including single drug or combined chemotherapy (33), such as anti-angiogenic drugs (such as bevacizumab, sunitinib), histone deacetylase inhibitors, as well as target-induced cell apoptosis drugs. The precise medicine currently is a hotspot of study area for patients with non-small cell lung cancer. The key of targeted therapy is to accurately analyze the pathological images. This analysis tool primarily depends on the clearly identifying pathological area by experienced pathologists. The visual inspection is time-consuming and subject to individual bias. Therefore, computer-aided diagnostic systems have developed rapidly for this clinical field, especially in the clinical application of multiple layer neural networks under deep learning. Deep learning networks can convert structured information, amid it can automatically identify and extract relevant features. However, there were also some challenges, such as important feature loss, over fitting, hyper-parameter adjustment, and other problems, which may affect the subsequent diagnosis and treatment design. Therefore, the application of deep neural networks on pathological images diagnosis has always been controversial.

Our study has some limitations. Firstly, TGCA database only includes cases in United States, which might result in ethnicity difference. Since the smokers are significantly higher in China than United States, therefore, the occurrence of lung cancer might cause by other influencing factors in addition of gene mutations. Moreover, the insufficient number of H&E-stained whole slide images we downloaded led to the imbalance of negative and positive samples which may affect the probability of some bio-markers. Moreover, non-specific features of H&E images, such as blood vessels, poorly stained areas, lung tissue necrosis areas, and overlapping blurred areas have been removed in our model, however, the blood vessels also indicate early metastasis, and staining technique may cause sample imbalance. Finally, our model did not include an independent verification subset to evaluate our model, which may have certain effects on our results.

## Methods

### Deep Convolution Gaussian Mixture Model

The deep convolution Gaussian mixture model (DCGMM) is an unsupervised clustering algorithm which is based on the Gaussian mixture algorithm combined with a deep convolution network. The Gaussian mixture algorithm means a probability distribution algorithm with the below form:

(1) $$P\left(y|\theta \right)=\overset{K}{\underset{k=1}{\sum }}\alpha_{k}\phi \left(y|\theta_{k}\right)$$

Here, α_k_ is the weight coefficient of each Gaussian distribution function, also known as the mixing coefficient, must satisfy 0≤ α_k_ ≤1 and $\overset{K}{\underset{k=1}{\sum }}\alpha_{k}=1$; *ϕ*(y|θ_k_) is the density of Gaussian distribution, and $\theta_{k}=\left(\mu_{k},\sigma_{k}^{2}\right)$. $\emptyset \left(y|\theta_{k}\right)=\frac{1}{\sqrt{2\pi }\sigma_{k}}\exp\left(-\frac{{\left(y-\mu_{k}\right)}^{2}}{2\sigma_{k}^{2}}\right)$ is K_th_ sub-model.

Gaussian Mixture Model (GMM) is widely used in many cases, moreover the expectation maximization (EM) algorithm is an effective method to learn the parameters in GMM. GMM is divided into two steps similar to K-means:

Step E: Estimate the probability which is generated by each sub-model. For each data x_i_, the probability generated by the k-th sub-model (that is, the responsiveness of the sub-model k to the appearance data x_i_) is

(2) $$\gamma \left(i,k\right)=\frac{\pi_{k}N(x_{i}|\mu_{k},\sum_{k})}{\sum_{j=1}^{K}\pi_{j}N(x_{i}|\mu_{j},\sum_{j})}$$

Step M: Based on the maximum likelihood estimation.

(3) $$\mu_{k}=\frac{1}{N_{k}}\overset{N}{\underset{i=1}{\sum }}\gamma \left(i,k\right)x_{i}$$

(4) $$\sum_{k}=\frac{1}{N_{k}}\overset{N}{\underset{i=1}{\sum }}\gamma \left(i,k\right)\left(x_{i}-\mu_{k}\right){\left(x_{i}-\mu_{k}\right)}^{T}$$

Where $N_{\text{k}}=\overset{\text{N}}{\underset{\text{i}=1}{\sum }}\text{γ}\left(\text{i},\text{k}\right)\;$and *π_k_* can also be estimated as N_k_/N.

From formula (1), the natural log-likelihood function could be represented as

(5) $$lnP\left(Y|\alpha ,\theta \right)=\overset{M}{\underset{m=1}{\sum }}ln\left\{\overset{K}{\underset{k=1}{\sum }}\alpha_{k}Æ(y|\theta_{k})\right\}$$

Here M is the total count of pixels in the input picture (Y = {y_1_, y_2_,…, y_M_}). Given GMM, the goal is finding targeted parameters (θ_k_, Σ_k_, α_k_). A common approach is using an EM algorithm to iteratively evaluate responsibilities [formula (3)] and re-estimate the parameters.

Some recent studies have made some new developments on traditional GMM. GMM can be applied to auto encoder neural networks for low-dimensional representations (34), or stacks of multiple GMM layers can be constructed on top of each other in a hierarchical architecture (35). Therefore, deep convolutional Gaussian mixture model (DCGMM) combines the parameters of the CNN with the parameters of the GMM to optimize the model.

The Deep Convolutional Gaussian Mixture Model (DCGMM) combines the Gaussian Mixture Model (GMM) into the color allocation of the image through the high image representation ability of the Convolutional Neural Network (CNN) to perform the color normalization of the image. We use convolutional neural networks to estimate the liability coefficient. Then the parameters of DCGMM are optimized by gradient descent method and log-likelihood function (Equation 5). In fact, DCGMM first replaces the E step in the EM algorithm with a convolutional neural network, and then uses the existing responsibility parameter estimation to estimate the θ and Σ of the multivariate Gaussian distribution like the M step in the EM algorithm. Finally, the training of DCGMM is mainly carried out through gradient descent method and back propagation.

### Convolutional Neural Networks

**Figure 6:**
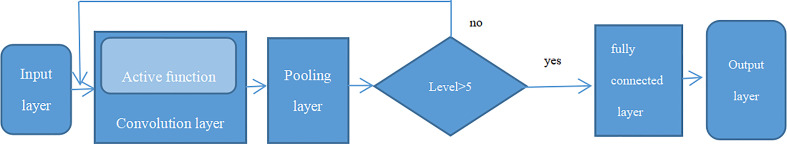
Convolutional neural network structure, A complete convolutional neural network basically includes five components: input layer, convolutional layer, pooling layer, fully connected layer and output layer.

Previous studies have shown that Convolutional Neural Networks (CNN) is the leading deep learning method for tumor diagnosis. The earliest application of neural network was a multilayer perceptron (MLP) with multiple levels of conversion. The traditional neural network contains input layer, hidden layers, and the output layer, and if it includes multiple hidden layers, it is known as a “deep neural network” (36). A complete convolutional neural network basically includes five components: input layer, convolutional layer, pooling layer, fully connected layer, and output layer, as seen in **Figure 6** (convolutional neural network structure, A complete convolutional neural network basically includes five components: input layer, convolutional layer, pooling layer, fully connected layer, and output layer). Firstly, the data came from input layer, then it was further input into convolutional layer which was the kernel content of the entire neural network especially in the sequential process. Generally, the convolution of node matrix from previous layer of the neural network is converted into node matrix on next layer of the neural network, and the depth of the node matrix is increased to achieve a deeper expression. The convolution layer generally has an excitation function to help express complex features through rectified linear unit (ReLu). The crucial function of the pooling layer is the extract features for dimensional reduction through ReLu excitation layer. The pooling sample layer does not change the depth of the feature matrix, but it can reduce the size of the matrix and simplify the constitution of the neural network. The fully connected layer is located after multiple convolution pooling processes to achieve the final classification result. The output layer is used to receive the probability distribution of the results. The training of convolutional neural network primarily contains forward propagation and back propagation. Among the training course, the parameters can be constantly changed in order to obtain greatest simulation effect. With increasing the deep of the exploration of convolutional neural networks, a series of optimized and improved structural models have emerged, such as fully convolutional neural networks, deep convolutional neural networks, etc. Deep convolutional neural network is a valid and steady mean for the image processing (37). In order to maintain the optimal pixels, shape, and other characteristics attribution of image, the deep convolutional neural network improves the network constitution through the local features and local perceptions, shared weights, spatial or temporal pool sampling (38).

Since the significance of the neural network studying process is to study the data allocation, once the apportion of training data is inconsistent to the test data, the generalization capacity of the network will sharply decrease. In addition, once the apportion of each heap of training data is inconsistent, the network must study to fit the inconsistent allocations in each iteration, that will significantly decrease the training rate of the network. Once the neural network starts training, the parameters will be changed. In addition to the input layer, the input data allocation of every layer of the subsequent network has been changed. During the training process, the updated of the training parameters from the former layer will cause the changes of input data in the latter layer. Although stochastic gradient descent (SGD) is simple and effective for training deep networks, it requires human settings and wastes time to adjust parameters for instance learning rate, weight attenuation coefficient, parameter initialization, dropout ratio, etc. (39). Moreover, the input of every layer is influenced by the parameters of all former layers, causing the layer ceaselessly adjust the novel allocation, which is, the distribution of internal nodes in the deep network during the training process (Internal Covariate Shift). If the allocation of training data keeps changing during the training process, it will slow down the training efficiency, and network will be deeper as well. Thereby, Batch Normalization (BN) layer in the convolutional neural network is recommended to improve the generalization capacity and expedite training process when data distribution of middle layer changes during the training process (40). By using normalization as part of the model architecture, normalization is performed for each training batch, which is, with inputting at each layer of the network, a normalization process is performed before entering the next layer of the network. The BN algorithm independently normalizes each scalar feature, which is, the mean is 0 and the variance is 1. The formula is as follows:

(6) $$x^{,k}=\frac{x^{k}-E\left[x^{k}\right]}{\sqrt{Var\left[x^{k}\right]}}$$

Where, E[x^k^] is supposed to the mean value of every batch of training data neurons x^k^, and the denominator is the standard deviation of the activation level of every batch of data neurons x^k^.

If only each input of the layer is normalized, it may change the representation of layers. Therefore, in order to maintain the identity transformation, we used transformation reconstruction and introduced the learn-able parameters γ and β, thereby:

(7) $$\begin{array}{l} y^{k}=\gamma^{k}x^{,k}+\beta^{k} \end{array}$$

When $\gamma^{k}=\sqrt{Var\left[x^{k}\right]},\beta^{k}=E\left[x^{k}\right]$ the data was restored back.

Therefore, we added a batch normalization (BN) layer to the CNN model, which would greatly increase the training rate and accuracy of the convolutional neural network.

### Residual Net

**Figure 7:**
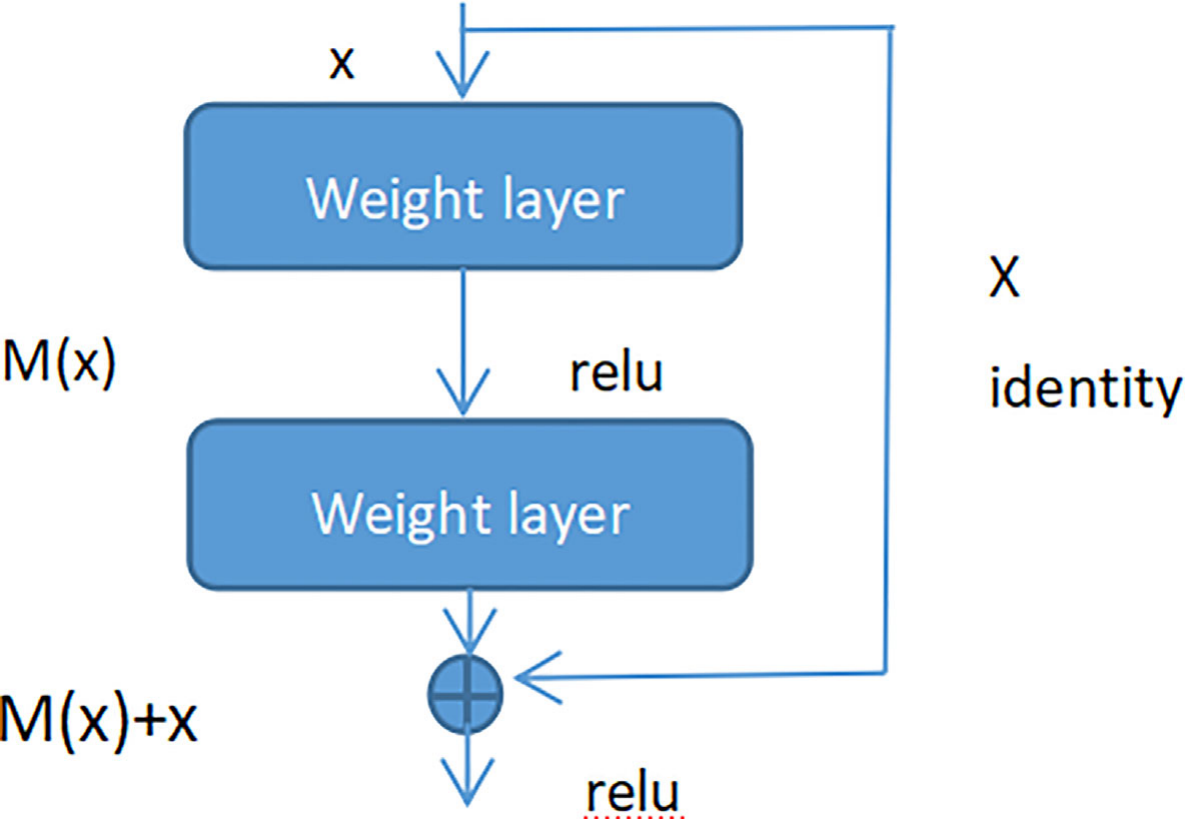
Residual block.

Adding batch normalization will grow the depth of the convolutional neural network. The depth of the deep learning has significant influence on the final classification and recognition. Therefore, the traditional idea is that with increased depth the network, the performance is better. But in fact, when the stacking of conventional networks (plain network) is deeper, the effect is worse. One of the reasons is that the network is deeper, it is more likely to cause the gradients disappear and gradients explode. However, the shallow network cannot significantly improve the recognition effect of the network, therefore, we introduced Res-Net to solve this problem (41). By adding shortcut connections, the residual network becomes easier to be optimized. Several layers of networks including the shortcut connection is called a residual block which is exhibited in **Figure 7**.

As seen in **Figure 7** (Residual block), x represented as the input, and M(x) represented as the output of the residual block before the activation function of the second layer, i.e. M(x) = W_2_/µ (W_1_ * x), where W_1_ and W_2_ respectively mean the weight of the first layer and the second layer, µ means the ReLu activation function. The output of final residual block was µ(M(x) + x).

As there is no shortcut link, the residual block is a common 2 layer network. The network in the residual block can be the fully connected layer or the convolutional layer. Suppose that the output of the second layer network precedes the activation function is N(x). If the output of layer 1 is the input x in the layer 2 network, then for the network no shortcut link, it should be updated to N(x) = x. For the network no shortcut link, which is the residual block, if we want the output to be x, only need to optimize M(x) = N(x)-x to 0. The optimization of the latter is much easier compare to the preceding formula. The residual network is composed of much residual blocks. For an assumption, a big neural network with an input of X and an output activation value of B[l]. If we add two more layers to initial network, the ultimate output result will be B[l + 2]. The two layers could become a residual module. The ReLU activation function is used throughout the network, and any activation values are bigger than or equal to 0. For big networks, whether the residual block is jointed to the middle or the end of the neural network will not influence the property of the network, and these residual blocks are relatively easy to learn the identity function. Moreover, they could increase the learning efficiency. As the activation function of the neuron, Relu defines the nonlinear output of the neuron after linear transformation w^T^x + b. Namely, for the input vector x from the foregone layer of the neural network into the neuron, the neuron applying the linear adjustment activation function will output max (0, w^T^x + b) to the rear layer of neurons or as the output of the entire neural network (depends on the site of the current neuron in the network institution).

**Figure 8:**
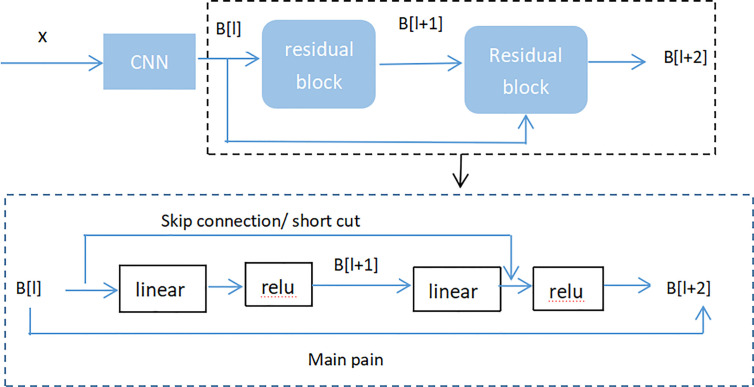
Residual neural network flow chart, showed two-layer residual neural network. The activation is performed on the L layer to obtain B[l+ 2].

**Figure 8** (Residual neural network flow chart, showed two-layer residual neural network. The activation is performed on the L layer to obtain B[l+ 2]) showed two-layer residual neural network. The activation is performed on the L layer to obtain B[l+ 1], and the activation was performed to gain B[L + 2]. And B[l + 2] = µ (D[l + 2] + C[l]), where D[l + 2] = E[l + 2] * B[l + 1] + C[l + 2], B[l + 1] = µ (D [l + 1]), D[l + 1] = E[l + 1] * B[l] + C[l + 1]. If E[l + 2] = 0 and C[l + 1] = 0, then we can get that B[l + 2] = µ (B[L]). When B[l]> = 0, B[l + 2] = B[l]. It is equivalent to establishing a linear relationship between B[l] and B[l + 2] when E and C are 0. Moreover, it is equivalent to directly copying the feature information of the B[l] layer to the B[l + 2] layer without affecting the network performance as well. For the residual network, as the network is deeper, the training error becomes smaller. This way could attain deeper layers of the network, which assists work out gradient disappearance and gradient explosion, allowing us to train deeper networks while ensuring good performance. In fact, the residual network consists of several shallow networks and does not fundamentally figure out the issue of gradient disappearance, instead to avoid the gradient disappearance. Since it composed of several shallow networks, the shallow network will not have the problem of disappearing gradients during the training process, but it can accelerate the convergence rate of the network.

### The Receiver Operating Characteristics

There are many ways to calculate the property of the model. Generally, the performance of the model is measured by the accuracy, recall, accuracy and F1 score, and the curve (AUC) under the receiver operating characteristics (ROC). This study used precision measurement receiver operating characteristics (ROC) to estimate the performance of the CNN model. The ROC curve was drawn *via* plotting the true positive rate (TPR) and false positive rate (FPR) under kinds of threshold putting, which is, the bight generated with FPR as the x axis and TPR as the y axis. The true positive rate (TPR) is also called sensitivity, that is the ratio of all actual positive samples that are exactly recognized as positive. Its expression is the same as expression of recall rate. The false positive rate (FPR) is also called specificity, which is the ratio of negative samples that are falsely identified as positive in actual negative samples. The strict mathematical definition is as follows:

(8) $$FPR=Sensitivity=\frac{FP}{TN+FP}$$

(9) $$TPR=Specificity=\frac{TP}{TP+FN}$$

Here, true positive (TP) is the count of samples that are forecast as positive and actually are positive, false positive (FP) is the count of samples that are forecast as positive and actually are negative. True negative (TN) is the count of samples that are forecast to be negative and really negative. False negative (FN) is the count of samples that are forecast to be negative and really is positive.

From the definition of FPR and TPR, it indicates the near the drawn ROC curve is to the upper left, the result is better. From a geometric angle of observe, with the bigger the region under the ROC curve, it indicates better model. Therefore, we used the area under the ROC curve, which is, AUC (Area Under Curve) value to measure performance of the model (42).

### Cross-validation

In order to conduct the reliable) and stable of CNN model, we used 2-fold cross-validation to increase the accuracy of the CNN algorithm by adjusting the hyper-parameters of the algorithm to gain the best scores. We split the data into two subsets, and one set was used as the training set to train data by CNN algorithm, and the other was used as a test set to predict the trained algorithm, thereby to find the error of the sample prediction, and summarize their squares afterwards. The above process was performed repeatedly until all samples were exactly predicted.

The hyper-parameters were adjusted which primarily included the number of filter cores, sample size, number of layers, and loss function of the CNN algorithm. For the adjustment of hyper-parameters, we firstly determined the activated function as Relu according to its mechanism, and determined the type of loss function and weight initialization afterward, and encoded method of the “output layer.” Secondly, according to the “broad strategy,” a simple structure was previously constructed to determine the number of “hidden layers” in the neural network and the number of neurons in each “hidden layer.” Thereby for the remaining hyper-parameters, we randomly selected a possible value, in order to adjust the learning rate without considering the regular terms in the loss function to reach a relatively appropriate threshold for the learning rate, then selected half of the threshold as the initial value. Then determine the size of the small batch of samples through experiments. Use the determined learning rate and verification data to select appropriate regularization parameters, and then return to re-optimize the learning rate. The overall observation of these experiments can determine learning rounds.

## Data Availability Statement

The original contributions presented in the study are included in the article/supplementary material. Further inquiries can be directed to the corresponding author.

## Author Contributions

BL and WZ conceived the concept of the work; KH, YY, and ZM collected or analyzed the data; KH performed literature search and designed the experiments; KH, and ZM wrote the paper; KH and F-XW modified and reviewed the manuscript. All authors contributed to the article and approved the submitted version. 

## Funding

This work was supported by the National Nature Science Foundation of China (Grant Nos 61863010, 11926205, 11926412, and 61873076), Natural Science Foundation of Hainan, China (Grant Nos. 119MS036), and 2019 Hainan Province Graduate Innovative Scientific Research Project (Hys2019-265).

## Conflict of Interest

The authors declare that the research was conducted in the absence of any commercial or financial relationships that could be construed as a potential conflict of interest.

## References

[1] Bray F Ferlay J Soerjomataram I Siegel RL Torre LA Jemal A. Global cancer statistics 2018: GLOBOCAN estimates of incidence and mortality worldwide for 36 cancers in 185 countries. CA: A Cancer J Clin (2018) 68:394–424. 10.3322/caac.21492 30207593

[2] Collins LG Haines C Perkel R Enck RE. Lung cancer: diagnosis and management. Am Fam. Physician (2007) 75:56–63. 10.1186/1471-2296-8-1 17225705

[3] Coudray N Ocampo PS Sakellaropoulos T Narula N Matija S Moreira DFAL. Classification and mutation prediction from non–small cell lung cancer histopathology images using deep learning. Nat Med (2018) 24:1559–67. 10.1038/s41591-018-0177-5 PMC9847512 30224757

[4] Travis WD Rekhtman N Riley GJ Geisinger KR Asamura H Brambilla E. Pathologic diagnosis of advanced lung cancer based on small biopsies and cytology: a paradigm shift. J Thorac Oncol (2010) 5:411–4. 10.1097/JTO.0b013e3181d57f6e 20357614

[5] Loo PS Thomas SC Nicolson MC Fyfe MN Kerr KM. Subtyping of undifferentiated non-small cell carcinomas in bronchial biopsy specimens. J Thorac Oncol (2010) 5:442–7. 10.1097/JTO.0b013e3181d40fac 20195168

[6] Travis WD Brambilla E Riely GJ. New pathologic classification of lung cancer: relevance for clinical practice and clinical trials. J Clin Oncol (2013) 31:992–1001. 10.1200/JCO.2012.46.9270 23401443

[7] Scagliotti G Hanna N Fossella F Sugarman K Blatter J Peterson P. The differential efficacy of pemetrexed according to NSCLC histology: a review of two phase III studies. Oncologist (2009) 14:253–63. 10.1634/theoncologist.2008-0232 19221167

[8] Sandler A Gray R Perry MC Brahmer J Schiller JH Dowlati A. Paclitaxel-carboplatin alone or with bevacizumab for non-small-cell lung cancer. N Engl J Med (2006) 355:2542–50. 10.1056/NEJMoa061884 17167137

[9] Yu KH Snyder M. Omics profiling in precision oncology. Mol Cell Proteomics (2016) 20:O116.059253. 10.1074/mcp.O116.059253 PMC4974334 27099341

[10] Snyder M. Genomics and Personalized Medicine: What Everyone Needs to Know. Oxford University Press (2016). 10.1093/wentk/9780190234775.001.0001

[11] Nicholson AG Gonzalez D Shah P Pynegar MJ Deshmukh M Rice A. Refining the diagnosis and EGFR status of non-small cell lung carcinoma in biopsy and cytologic material, using a panel of mucin staining, TTF-1, cytokeratin 5/6, and P63, and EGFR mutation analysis. J Thorac Oncol (2010) 5:436–41. 10.1097/JTO.0b013e3181c6ed9b 20068475

[12] Harpole DH Jr. Herndon JE 2nd Wolfe WG Iglehart JD Marks JR. A prognostic model of recurrence and death in stage I non-small cell lung cancer utilizing presentation, histopathology, and oncoprotein expression. Cancer Res (1995) 55:51–6. 10.1038/ajg.2012.444 7805040

[13] Yoshizawa A Motoi N Riely GJ Sima CS Gerald WL Kris MG. Impact of proposed IASLC/ATS/ERS classification of lung adenocarcinoma: prognostic subgroups and implications for further revision of staging based on analysis of 514 stage I cases. Mod Pathol (2011) 24:653–64. 10.1038/modpathol.2010.232 21252858

[14] Franklin WA. Diagnosis of lung cancer: pathology of invasive and preinvasive neoplasia. Chest (2000) 117:80S–9S. 10.1378/chest.117.4_suppl_1.80S 10777460

[15] Kerr KM. Personalized medicine for lung cancer: new challenges for pathology. Histopathology (2012) 60:531–46. 10.1111/j.1365-2559.2011.03854.x 21916947

[16] Stang A Pohlabeln H Muller KM Jahn I Giersiepen K Ckel KHJ. Diagnostic agreement in the histopathological evaluation of lung cancer tissue in a population-based case-control study. Lung Cancer (2006) 52:29–36. 10.1016/j.lungcan.2005.11.012 16476504

[17] Grilley-Olson JE Hayes N Moore DT Leslie KO Wilkerson MD Qaqish BF. Validation of interobserver agreement in lung cancer assessment: hematoxylin-eosin diagnostic reproducibility for non-small cell lung cancer: the 2004 World Health Organization classification and therapeutically relevant subsets. Arch Pathol Lab Med (2013) 137:32–40. 10.5858/arpa.2012-0033-OA 22583114 PMC5787023

[18] Raab SS Grzybicki DM Janosky JE Zarbo RJ Meier FA Jensen C. Clinical impact and frequency of anatomic pathology errors in cancer diagnoses. Cancer (2005) 104:2205–13. 10.1002/cncr.21431 16216029

[19] Zeng X Zhong Y Lin W Zou Q. Predicting Disease-associated Circular RNAs Using Deep Forests Combined with Positive-Unlabeled Learning Methods. Briefings Bioinf (2020) 21(4):1425–36. 10.1093/bib/bbz080 31612203

[20] Lv Z Ao C Zou Q. Protein Function Prediction: From Traditional Classifier to Deep Learning. Proteomics (2019) 19(14):e1900119. 10.1002/pmic.201900119 31187588

[21] Greenspan H van Ginneken B Summers RM. Guest editorial deep learning in medical imaging: overview and future promise of an exciting new technique. IEEE Trans Med Imaging (2016) 35:1153–9. 10.1109/TMI.2016.2553401

[22] Khosravi P Kazemi E Imielinski M Elemento O Hajirasouliha I. Deep Convolutional Neural Networks Enable Discrimination of Heterogeneous Digital Pathology Images. EBioMedicine (2018) 27:317–28. 10.1016/j.ebiom.2017.12.026 PMC5828543 29292031

[23] Kather JN Pearson AT Halama N Jaeger D Krause J Loosen SH. Deep learning can predict microsatellite instability directly from histology in gastrointestinal cancer. Nat Med (2019) 25:1054–6. 10.1038/s41591-019-0462-y PMC7423299 31160815

[24] Zhang Z Chen P McGough M Xing F Wang C Bui M. Pathologist-level interpretable whole-slide cancer diagnosis with deep learning. Nat Mach Intell (2019) 1:236–45. 10.1038/s42256-019-0052-1

[25] Lai GGY Lim TH Lim J Liew PJR Wang XLK Nahar R. Clonal MET Amplification as a Determinant of Tyrosine Kinase Inhibitor Resistance in Epidermal Growth Factor Receptor–Mutant Non–Small-Cell Lung Cancer[J]. J Clin Oncol (2019) 37:855–57. 10.1200/JCO.18.00177 30676858

[26] Noonan SA Berry L Lu X Gao D Baron AE Chesnut P. Identifying the appropriate FISH criteria for defining MET copy number-driven lung adenocarcinoma through oncogene overlap analysis. J Thorac Oncol (2016) 11:1293–304. 10.1016/j.jtho.2016.04.033 PMC5404374 27262212

[27] Lu S Fang J Li X Cao L Zhou J Guo Q. Phase II study of savolitinib in patients (pts) with pulmonary sarcomatoid carcinoma (PSC) and other types of non-small cell lung cancer (NSCLC) harboring MET exon 14 skipping mutations (METex14+). ASCO(9519) (2020) 21:317–466. 10.1200/JCO.2020.38.15_suppl.9519

[28] Helsten T Elkin S Arthur E Tomson BN Carter J Kurzrock R. The FGFR Landscape in Cancer: Analysis of 4,853 Tumors by Next-Generation Sequencing. Clin Cancer Res (2016) 22:259–67. 10.1158/1078-0432.CCR-14-3212 26373574

[29] Katoh M. Fibroblast growth factor receptors as treatment targets in clinical oncology. Nat Rev Clin Oncol (2019) 16:105–22. 10.1038/s41571-018-0115-y 30367139

[30] Fakih M O’Neil B Price TJ Falchook GS Desai J Kuo J. Phase 1 study evaluating the safety, tolerability, pharmacokinetics (PK), and efficacy of AMG 510, a novel small molecule KRASG12C inhibitor, in advanced solid tumors. J Clin Oncol (2019) 37:suppl; abstr 3003. 10.1200/JCO.2019.37.15_suppl.3003

[31] Zhang X Zhou Y Gu YE. Tanshinone IIA induces apoptosis of ovarian cancer cells in vitro and in vivo through attenuation of PI3K/AKT/JNK signaling pathways. Oncol Lett (2019) 17:1896–902. 10.3892/ol.2018.9744 PMC6341594 30675253

[32] Crabb SJ Birtle AJ Martin K Downs N Ratcliffe I Maishman T. ProCAID: a phase I clinical trial to combine theAKT inhibitor AZD5363 with docetaxel and prednisolone chemotherapy formetastatic castration resistant prostate cancer. Investigational New Drugs (2017) 35:599–607. 10.1007/s10637-017-0433-4 28144789 PMC5613074

[33] Wang Y Shenglong X Bin H. Effect of EGFR gene polymorphism on efficacy of chemotherapy combined with targeted therapy for non-small cell lung cancer in Chinese patients. Am J Cancer Res (2019) 9(3):619–27. PMC6448055 30949415

[34] Zong B Song Q Min MR Cheng W Lumezanu C Cho D. Deep autoencoding gaussian mixture model for unsupervised anomaly detection. In: In International Conference on Learning Representations. ICLR 2018 conference (2018) 11367:665–74.

[35] van den Oord A Schrauwen B. Factoring variations in natural images with deep gaussian mixture models. In: In Advances in Neural Information Processing Systems 27. Curran Associates, Inc. (2014). 2:3518–26.

[36] Litjens G Kooi T Bejnordi BE Setio AAA Ciompi F Ghafoorian M. A Survey on Deep Learning in Medical Image Analysis. Med Image Anal arXiv preprint arXiv:1702.05747 (2017) 42:60–88. 10.1016/j.media.2017.07.005 28778026

[37] Higgins JP Kaygusuz G Wang L Montgomery K Mason V Zhu SX. Placental s100 (s100p) and gata3: markers for transitional epithelium and urothelial carcinoma discovered by complementary dna microarray. Am J Surg Pathol (2007) 31:673–80. 10.1097/01.pas.0000213438.01278.5f 17460449

[38] Hinton GE Osindero S Teh Y-W. A fast learning algorithm for deep belief nets. Neural Comput (2006) 18:1527–54. 10.1162/neco.2006.18.7.1527 16764513

[39] Hinton GE Salakhutdinov RR. Reducing the dimensionality of data with neural networks. Science (2006) 313:504–7. 10.1126/science.1127647 16873662

[40] Wang J Li S An Z Jiang X Qian W Ji S. Batch-normalized deep neural networks for achieving fast intelligent fault diagnosis of machines. Neurocomputing (2019) 329:53–65. 10.1016/j.neucom.2018.10.049

[41] He K Zhang X Ren S Sun J. Deep Residual Learning for Image Recognition[C]// IEEE Conference on Computer Vision & Pattern Recognition. IEEE Comput Soc (2016) 5(2):157–66. 10.1109/CVPR.2016.90

[42] Zawistowski M Sussman JB Hofer TP Bentley D Hayward RA Wiitala WL. Corrected roc analysis for misclassifified binary outcomes. Stat Med (2017) 36:2148–60. 10.1002/sim.7260 28245528

